# 1080. Relebactam Increases Imipenem Activity Against Imipenem-Nonsusceptible and -Susceptible *Pseudomonas aeruginosa* and Enterobacterales: Assessment of Isolates from RESTORE-IMI 2

**DOI:** 10.1093/ofid/ofab466.1274

**Published:** 2021-12-04

**Authors:** David W Hilbert, C Andrew DeRyke, Maria C Losada, Pamela Moise, Luke F Chen, Katherine Young

**Affiliations:** 1 Merck & Co., Inc., Kenilworth, New Jersey; 2 Merck Research Labs, Merck & Co., Inc., Kenilworth, New Jersey; 3 Merck & Co, Inc, Kenilworth, NJ

## Abstract

**Background:**

Relebactam (REL) inhibits class A and C β-lactamases (BLs) and is approved in the US in the combination imipenem/cilastatin/REL (IMI/REL) for hospital acquired bacterial pneumonia (HABP) and ventilator associated bacterial pneumonia (VABP), and also in patients with limited treatment options for complicated urinary tract infections and complicated intraabdominal infections. The objective of this study was to evaluate the potentiation of imipenem (IMI) by REL in baseline respiratory isolates from the recently completed Phase 3 RESTORE-IMI 2 study that demonstrated efficacy and safety of IMI/REL in the treatment of patients with HABP/VABP.

**Methods:**

Baseline lower respiratory tract (LRT) isolates were evaluated for IMI MICs in the presence and absence of REL using broth microdilution and CLSI interpretive criteria. All *Pseudomonas aeruginosa* and Enterobacterales for which IMI/REL is either indicated or the MIC_90_ is less than or equal to the susceptibility breakpoint were evaluated.

**Results:**

Summary statistics and the MIC distribution for *P. aeruginosa* are shown in the figure. For *P. aeruginosa*, REL reduces the IMI mode MIC of IMI-nonsusceptible (IMI-NS) (MIC >2) isolates ≥8-fold (from 16-32 to 2 µg/mL) and that of IMI-susceptible (IMI-S) (MIC ≤2) isolates ≥2-fold (from 1 to ≤0.5 µg/mL). Among Enterobacterales, the IMI mode MIC of IMI-NS (MIC >1) isolates was reduced ≥4-fold (from 2 to ≤0.5 µg/mL). REL enhanced the activity of IMI among IMI-S isolates (MIC ≤1), most notably observed in Enterobacterales species that produce a chromosomal AmpC, increasing the proportion with MIC ≤0.5 µg/mL from 76% to 98%.

MIC Distribution and Summary Statistics of RESTORE IMI-2 Isolates

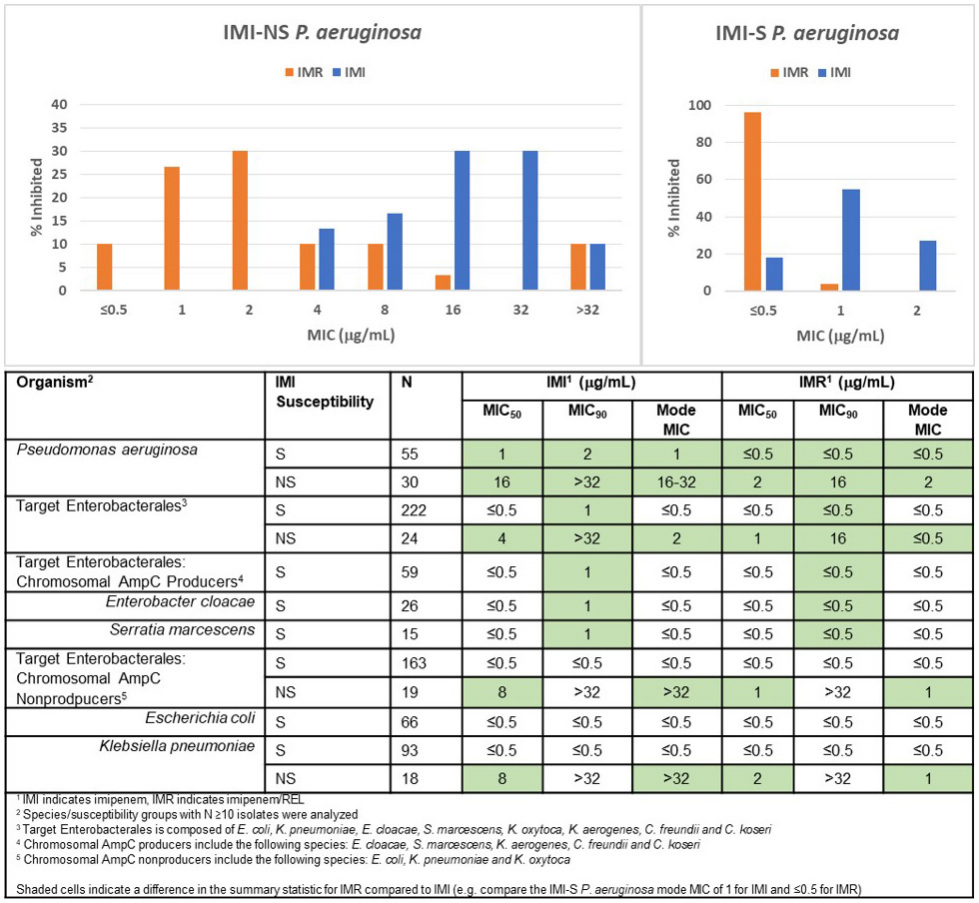

**Conclusion:**

Among baseline LRT isolates from RESTORE-IMI 2 the potentiation of IMI by REL results in the restoration of susceptibility among IMI-NS *P. aeruginosa* and Enterobacterales and enhanced IMI activity among IMI-S isolates. This enhanced activity among IMI-S Enterobacterales is most notable among species with reported chromosomal expression of AmpC. This lowering of IMI MICs upon addition of REL contributes to the high probability of target attainment (≥90%) observed following administration of IMI/REL 1.25g every 6 hours, further supporting the IMI/REL efficacy data observed in RESTORE-IMI 2.

**Disclosures:**

**David W. Hilbert, PhD**, **Merck** (Employee) **C. Andrew DeRyke, PharmD**, **Merck & Co., Inc.** (Employee, Shareholder) **Maria C. Losada, BA**, **Merck** (Employee) **Pamela Moise, PharmD**, **Merck** (Employee) **Luke F. Chen, MBBS MPH MBA FRACP FSHEA FIDSA**, **Merck** (Employee) **Katherine Young, MS**, **Merck** (Employee)

